# Integrin αvβ6 as a Target for Tumor-Specific Imaging of Vulvar Squamous Cell Carcinoma and Adjacent Premalignant Lesions

**DOI:** 10.3390/cancers13236006

**Published:** 2021-11-29

**Authors:** Bertine W. Huisman, Merve Cankat, Tjalling Bosse, Alexander L. Vahrmeijer, Robert Rissmann, Jacobus Burggraaf, Cornelis F. M. Sier, Mariette I. E. van Poelgeest

**Affiliations:** 1 Center for Human Drug Research, 2333 CL Leiden, The Netherlands; bhuisman@chdr.nl (B.W.H.); mcankat@chdr.nl (M.C.); rrissmann@chdr.nl (R.R.); kb@chdr.nl (J.B.); m.i.e.van_poelgeest@lumc.nl (M.I.E.v.P.); 2 Department of Gynecology, Leiden University Medical Center, 2333 ZA Leiden, The Netherlands; 3 Department of Pathology, Leiden University Medical Center, 2333 ZA Leiden, The Netherlands; t.bosse@lumc.nl; 4 Department of Surgery, Leiden University Medical Center, 2333 ZA Leiden, The Netherlands; a.l.vahrmeijer@lumc.nl; 5 Leiden Academic Center for Drug Research, Leiden University, 2333 CC Leiden, The Netherlands; 6 Percuros BV, 2333 CL Leiden, The Netherlands

**Keywords:** vulva, squamous carcinoma, HSIL, dVIN, fluorescence-guided surgery, integrin αvβ6

## Abstract

**Simple Summary:**

Vulvar tumors are sometimes difficult to distinguish from adjacent healthy tissue during surgery, causing recurrence rates of up to 40% and co-morbidity. Fluorescence-guided surgery illuminating neoplastic tissue is increasingly being used to assist surgeons for various types of cancers. As no suitable tracers are known yet for vulvar tumors, we have evaluated which proteins could be targeted for fluorescence-guided surgery. Immunohistochemistry was used to study the distribution of nine membrane proteins in healthy and (pre)malignant tissues that were consequently analyzed using quantitative image analysis. Integrin αvβ6 allowed for the most robust discrimination of VSCCs and adjacent premalignant lesions compared with surrounding healthy tissue. The use of an αvβ6 targeted near-infrared fluorescent probe for FGS of vulvar (pre)malignancies should be evaluated in future studies.

**Abstract:**

Surgical removal of vulvar squamous cell carcinoma (VSCC) is associated with significant morbidity and high recurrence rates. This is at least partially related to the limited visual ability to distinguish (pre)malignant from normal vulvar tissue. Illumination of neoplastic tissue based on fluorescent tracers, known as fluorescence-guided surgery (FGS), could help resect involved tissue and decrease ancillary mutilation. To evaluate potential targets for FGS in VSCC, immunohistochemistry was performed on paraffin-embedded premalignant (high grade squamous intraepithelial lesion and differentiated vulvar intraepithelial neoplasia) and VSCC (human papillomavirus (HPV)-dependent and -independent) tissue sections with healthy vulvar skin as controls. Sections were stained for integrin αvβ6, CAIX, CD44v6, EGFR, EpCAM, FRα, MRP1, MUC1 and uPAR. The expression of each marker was quantified using digital image analysis. H-scores were calculated and percentages positive cells, expression pattern, and biomarker localization were assessed. In addition, tumor-to-background ratios were established, which were highest for (pre)malignant vulvar tissues stained for integrin αvβ6. In conclusion, integrin αvβ6 allowed for the most robust discrimination of VSCCs and adjacent premalignant lesions compared to surrounding healthy tissue in immunohistochemically stained tissue sections. The use of an αvβ6 targeted near-infrared fluorescent probe for FGS of vulvar (pre)malignancies should be evaluated in future studies.

## 1. Introduction

Vulvar squamous cell carcinoma (VSCC) is a rare type of cancer with an incidence of 1.5–2.7 per 100,000 women, necessitating specialized centralized care [1]. Overall incidence worldwide is increasing. For the Dutch population, this increase is mainly observed in younger women [2,3]. There are two major pathways for the development of VSCC. Human papilloma virus (HPV)-dependent VSCC (20%) are caused by high-risk HPV types, predominantly HPV16. HPV-dependent VSCC arises in the background of precursor lesions named high grade squamous intraepithelial neoplasia (HSIL). Another precursor of VSCC is differentiated vulvar intraepithelial neoplasia (dVIN), which often arises in elderly women with lichen sclerosus (LS), a chronic inflammatory skin disease. For dVIN, the cumulative risk of malignant progression to VSCC is estimated to be as high as 50% after 10 years and this type of VSCC has a poor survival compared to HPV-dependent VSCC [1,4,5,6]. The cornerstone of treatment for VSCC consists of surgery with or without radiochemotherapy [7,8]. Even in early-stage disease, the recurrence rate is up to 40% after 10 years, requiring repeated local surgery or (re)irradiation [9].

In more than half of the VSCC patients, surgery in the vulvar area, in particular when the tumor is located near the urethra, clitoris, or anus, is associated with significant morbidity, including disfigurement, sexual dysfunction, and psychological problems [10]. These morbidities are partly caused by the limited ability to distinguish between healthy and (pre)malignant tissue during surgery. The recognition and excision of vulvar lesions relies on the gynecologist’s visual and tactile skills, experience, and information obtained from histological biopsies. Recognition by the gynecologist is even harder when the vulvar architecture is complicated by deformation associated with inflammation, atrophy, or previous treatments [2,11]. Positive surgical margins are associated with higher risk for local recurrence and poor survival [12,13]. In addition, precursor lesions are often found adjacent to the tumor, which are sometimes difficult to identify clinically and therefore not treated adequately. Consequently, better identification and timely recognition of vulvar (pre)malignant lesions may result in prevention of re-excisions, local recurrences, metastases, and associated prognosis. Real time visualization during fluorescence-guided surgery (FGS) could aid in resolving this problem.

FGS is a promising technique for real-time detection of occult tumor lesions and localization of cancer margins. The procedure makes use of a cell-specific targeting agent, linked to a near-infrared fluorescent (NIRF) dye or radiolabel, which can be visualized in real time by an advanced imaging system. Clinical studies on various cancer types have shown that FGS improves the recognition of tumor tissue significantly, primarily in cases with incomplete visual and tactile information [14,15]. Proper identification of tumor-specific targets for molecular imaging is key to the success of FGS [16]. In the last decade, FGS targets have been explored for cancer types including ovarian, colorectal, and head-and-neck cancer [17,18,19,20,21]. Until now, potential targets for FGS in VSCC have not been studied.

The aim of this study was to examine the expression of previously identified VSCC-specific membrane-associated targets based on the available literature and candidate targets for other squamous cancers in dVIN, HSIL, and VSCC tissues. To determine their suitability as a target for tumor-specific imaging in vulvar (pre)malignancies. The markers assessed are integrin alphavbeta6 (αvβ6), carbonic anhydrase IX (CAIX), CD44 variant 6 (CD44v6), epidermal growth factor receptor (EGFR), epithelial cell adhesion molecule (EpCAM), folate receptor α (FRα), multidrug resistance-associated protein (MRP1), mucin 1 (MUC1), and urokinase plasminogen activator receptor (uPAR) [17,18,20,22,23,24]. 

## 2. Materials and Methods

### 2.1. Tissue Samples

Pretreatment VSCC samples and precursor lesions, i.e., dVIN and HSIL (without information on treatment status) were collected from the pathology department of Leiden University Medical Center. Sample selection was based on original diagnosis described in the pathology report and the size of the available tissues. Non-squamous vulvar cancers were excluded. Formalin fixed paraffin embedded (FFPE) HPV-negative vulvar tissue from healthy anonymized women who underwent labia reduction surgery was included as control. Sample collection was approved by the local ethics review board (Medische Ethische Toetsingscommissie Leiden Den Haag Delft-reference number B19.025). All routinely made hematoxylin–eosin-(H&E), p16-(overexpressed in dysplastic tissue related to HPV infection; HSIL and HPV-dependent VSCC tissues) and p53-stained slides were reviewed by an expert gynecologic pathologist (TB) blinded to immunohistochemical results and lesions diagnosed and delineated [25,26,27,28]. Intentionally, fifteen samples were collected of each vulvar tissue type/group, i.e., healthy, dVIN, HPV-independent VSCC, HSIL and HPV-dependent VSCC. Some samples were excluded because the original diagnosis of a tissue could not be confirmed or (pre)malignant cells were no longer present in the tissue sample.

### 2.2. Immunohistochemistry

FFPE tissue blocks were sectioned into tissue sections of 4 µm. Sections were deparaffinized in xylene and rehydrated via serially diluted ethanol solutions. Endogenous peroxide was blocked for 20 min with 0.3% hydrogen peroxide diluted in demineralized water. Appropriate antigen retrieval was performed depending on the antibody (Table S1). Subsequently, sections were incubated overnight at room temperature (RT) with the primary antibodies. The optimal dilution for each of the antibodies was determined beforehand on vulvar normal and/or squamous cell carcinoma test tissue. Slides were washed three times with phosphate-buffered saline (PBS, pH 7.5) before 30 min incubation at RT with the appropriate secondary antibody, followed by another washing step. Staining was visualized with 3,3-diaminobenzidine tetrahydrochloride solution (DAB, K3468, Agilent Technologies, Inc., Santa Clara, CA, USA) for approximately 5 min at RT and counterstained for 20 s with hematoxylin (4085.9002, VWR International, Amsterdam, The Netherlands). After dehydration, the slides were mounted with Pertex (0081EX, Histolab, Askim, Sweden). Control staining’s were performed (Table 1 and Figure S1).

### 2.3. Digital Pathology Image Analysis

Stained sections were digitalized with a Panoramic Digital Slide Scanner (3D Histech, Budapest, Hungary), stored as tiled tiff format, and imported into QuPath (version 2.0.0). QuPath is an open-source software tool for digital pathology image analysis [29,30,31]. Based on delineation of tissues by pathologist TB, tumor and premalignant borders were manually annotated in Qupath by BH and MC and copied to sequential sections. Within all these tissue annotations, cell detection, cell classification and staining quantification was conducted by a script (Script S1). After running the script, an export file of the results was automatically generated in MS excel. This export file included intensity thresholds for positive stained cells, divided in three categories: low (1+), medium (2+) or high (3+) intensity staining (examples of by QuPath processed image are shown in Figures S2 and S3).

### 2.4. Marker Staining

Based on the number of positive cells and their intensity, Qupath automatically generated H-scores by the formula ‘1 × (% cells threshold 1+) + 2 × (% cells threshold 2+) + 3 × (% cells threshold 3+)’ for all annotations. H-scores range from 0 to 300, giving more relative weight to higher-intensity staining in a tissue section. The following H-score categories were defined: 0–50 low, 50–250 medium and 250–300 high marker staining [32]. Median, minimum and maximum H-scores per vulvar tissue type were calculated. In addition, tumor-to-background ratios (TBRs) were calculated by dividing the median H-score of vulvar (pre)malignant tissue by the median H-score for healthy vulvar tissue.

### 2.5. FGS Criteria

A potential protein marker for FGS based on IHC-staining was defined by fulfillment of all the following criteria [16,22,33]:

- a median H-score in (pre)malignant tissue being at least twice as high as the median H-score in healthy control and stromal tissue [34];
- a minimum median H-score in (pre)malignant tissue of at least 25;
- homogeneous expression throughout the tumor;
- cell surface protein expression.

### 2.6. Statistics

Median, minimum, and maximum H-scores were extracted from Qupath and TBRs calculated (Table 2). To test for favorable TBRs, a statistical analysis was performed on the comparison of median H-scores per vulvar tissue types (healthy vulva (HV)/dVIN, HV/HPV-independent VSCC, HV/HSIL, HV/HPV-dependent VSCC, dVIN/HPV-independent VSCC, HSIL/HPV-dependent VSCC) using a Mann–Whitney U test. Statistical analyses were performed using Graphpad Prism version 9.1.0 for MacOS (Graphpad Prism Software, San Diego, CA, USA). These results were presented in boxplots with 1st and 3rd quartiles. Several tissue sections contained different tissue type annotations located at one section. Only H-scores of annotations of the predefined tissue type of a patient were included in this analysis. Annotations located near the predefined tissue type annotation (e.g., VSCC patient with adjacent healthy tissue) were not included. As the data were not expected to be normally distributed, a Mann–Whitney U test was used to test for statistical significance of difference in group medians. No adjustment was considered for multiple testing issues due to the exploratory nature of this study. Thus, hypothesis testing results with *p* < 0.05 were considered statistically significant. For marker(s) that showed potential as FGS target based on the selection criteria (Section 2.5), a spaghetti plot was generated for data visualization. The lines in this plot represent patients, the dots are average H-scores per vulvar tissue type within a VSCC patients tissue section. This plot was completed for both HPV-dependent and -independent VSCC patients. No statistical analysis was performed; these data were used for visualization of H-scores and corresponding TBRs per patient.

## 3. Results

### 3.1. Tissue Characteristics

In total, 10 dVIN, 16 HPV-independent VSCC, 15 HSIL, 13 HPV-dependent VSCC tissues and 15 healthy vulvar controls were included for biomarker expression evaluation. Due to incidental poor slide quality, not all selected tissue samples could be scored for each marker.

### 3.2. Immunohistochemical Marker Staining

Hereafter, a narrative description of the staining of each marker will be given categorized by the previously mentioned four FGS criteria (Section 2.5). Markers are listed in alphabetic order.

#### 3.2.1. αvβ6—Integrin Alphavbeta6

**Figure 1:**
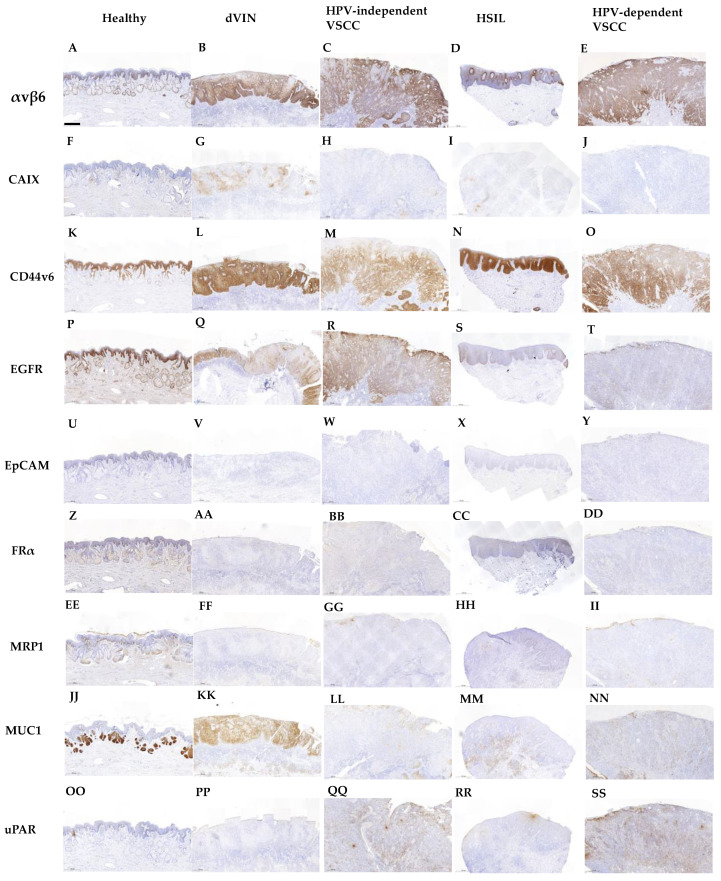
Representative images of αvβ6 (**A**–**E**), CAIX (**F**–**J**), CD44v6 (**K**–**O**), EGFR (**P**–**T**), EpCAM (**U**–**Y**), FRα (**Z**–**DD**), MRP1 (**EE**–**II**), MUC1 (**JJ**–**NN**) and uPAR (**OO**–**SS**) expression in healthy vulvar tissue with (sebaceous) glands, differentiated vulvar intraepithelial neoplasia (dVIN), human papilloma virus-independent vulvar squamous cell carcinoma (HPV-independent VSCC), high grade squamous intraepithelial lesion (HSIL) and human papilloma virus-dependent vulvar squamous cell carcinoma (HPV-dependent VSCC). All images show only the predefined tissue type of that section (no adjacent tissue). Scale bars represent 500 µm.

**Figure 2:**
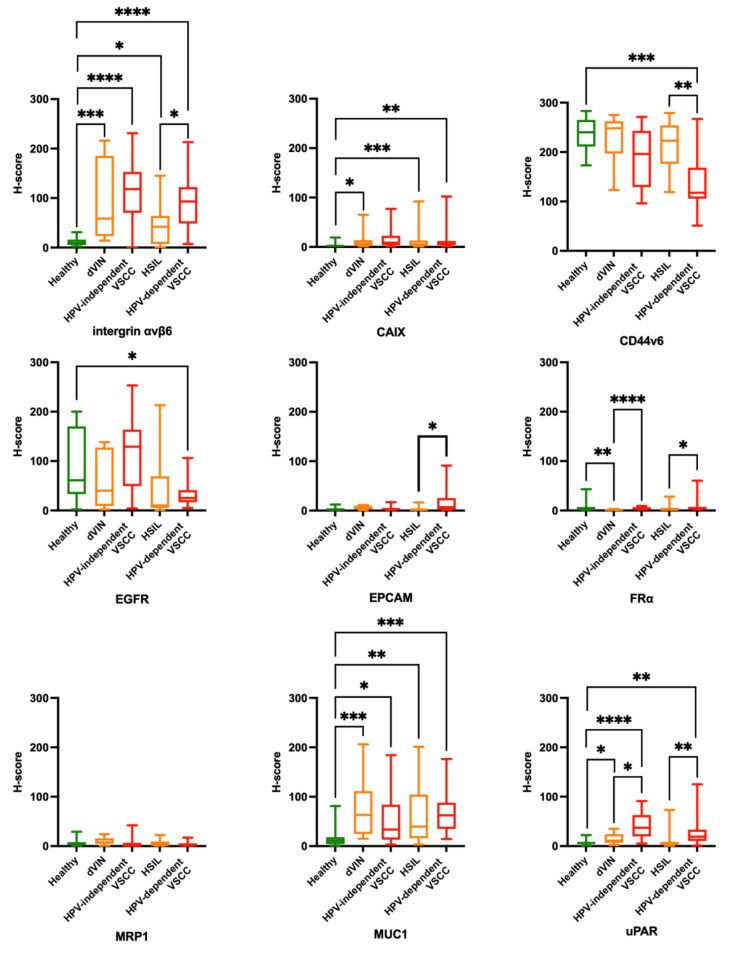
Boxplots representing median H-scores per vulvar tissue type (including 1st and 3rd percentiles) for integrin αvβ6, CAIX, CD44v6, EGFR, EpCAM, FRα, MRP1, MUC1, uPAR. Statistical analyses were performed between different median H-scores: HV/dVIN, HV/HPV-independent VSCC, HV/HSIL, HV/HPV-dependent VSCC, dVIN/HPV-independent VSCC, HSIL/HPV-dependent VSCC. ns = *p* > 0.05 (not shown), * = *p* ≤ 0.05, ** = *p* ≤ 0.01, *** = *p* ≤ 0.001, **** = *p* ≤ 0.0001. 10 dVIN, 16 HPV-independent VSCC, 15 HSIL, 13 HPV-dependent VSCC tissues and 15 healthy vulvar controls were included.

- Stromal tissue lacked αvβ6 expression. Healthy vulvar epithelium showed no or low expression of αvβ6. If αvβ6 was present in healthy vulvar tissue, it was mainly located in the spinosal and basal layer of the epithelium (Figure 1A). In addition, αvβ6 expression was higher in normal vulvar tissues wherein sebaceous glands were present (11/15 healthy vulvar tissues) compared with vulvar tissue sections that lacked those glands. αvβ6 staining within sebaceous glands was low to moderate (Figure 1A). The median H-score of healthy vulvar tissue was significantly lower compared with median H-scores of all vulvar (pre)malignant tissue types (Figure 2), resulting in TBRs > 2 (Table 2).

- Moderate αvβ6 expression was observed in 4/8 dVIN, 14/16 HPV-independent VSCC, 4/13 HSIL and 10/13 HPV-dependent VSCC tissues (Figure 1B–E respectively). αvβ6 expression lacked in 2/16 HPV-independent VSCC and 3/13 HPV-dependent VSCC tissues. The other premalignant tissues showed low expression. More intense αvβ6 staining was found in HSIL adjacent to HPV-dependent VSCC (average H-score 42) compared with isolated HSIL (average H-score 114). This difference was not observed for dVIN. Median H-scores per vulvar (pre)malignant tissues type were all above 25 (Table 2).
- αvβ6 was homogeneously expressed in all HPV-independent VSCC tissues. 2/10 HPV-dependent VSCC tissues showed a patchy staining pattern throughout the tumor, for 2/10 expression was restricted to the spinosal and/or basal layers, the remainder showed homogeneous expression. To a greater or lesser extent in all dVIN and HSIL tissues, as for healthy vulvar tissue, αvβ6 expression was restricted to the spinosal and/or basal layers (Figure 1A,D).
- αvβ6 showed cell membrane staining.

#### 3.2.2. CAIX—Carbonic Anhydrase IX

- Stromal and healthy vulvar epithelium lacked CAIX staining (Figure 1F). The median H-score of healthy vulvar tissues was significantly lower compared with median H-scores of dVIN, HSIL and HPV-dependent VSCC tissue groups (Figure 2), resulting in TBRs > 2 (Table 2). The median H-score of HPV-independent VSCC tissue group was not tested significantly higher compared with the median H-score of healthy vulvar tissue (TBR 4.5, Table 2).
- Most vulvar (pre)malignant tissues showed low CAIX expression (Figure 1H–J), 1–2 samples per tissue group showed moderate CAIX expression (Figure 1G). Median H-scores per vulvar (pre)malignant tissues type were all below 25 (Table 2).
- If CAIX staining was observed, it was positioned in the spinosal and/or basal layers of the vulvar epidermis in a heterogeneous and patchy pattern (Figure 1G–J).
- CAIX showed cell membrane staining.

#### 3.2.3. CD44v6—CD44 Variant 6

- Stromal tissue lacked CD44v6 staining. Healthy vulvar epithelium showed in 7/15 tissues high CD44v6 expression, the remaining tissues showed moderate expression (Figure 1K). TBRs were inverse for all (pre)malignant vulvar tissue types, indicating downregulation of CD44v6 in (pre)malignant compared with healthy tissue (Figure 2). Consequently, TBRs were not in favor for FGS application at the surface of the vulva (Table 2).
- Predominantly moderate CD44v6 staining was observed in vulvar (pre)malignant tissues (Figure 1L–O), in 5/10 dVIN, 3/16 HPV-independent VSCC, 5/15 HSIL and 1/12 HPV-dependent VSCC tissues high CD44v6 expression was observed. Median CD44v6 H-scores per vulvar (pre)malignant tissues type were all above 25 (Table 2).
- CD44v6 showed homogenous expression.
- CD44v6 showed cell membrane staining.

#### 3.2.4. EGFR—Epithelial Cell Adhesion Molecule

- EGFR staining was observed in glands, blood vessels and adnexa. Healthy vulvar epithelium showed moderate EGFR expression in 10/15 tissues (Figure 1P) and low expression in 5/15 tissues. TBRs were inverse for all (pre)malignant vulvar tissue types, indicating downregulation of EGFR in (pre)malignant tissue compared with healthy (Figure 2) Consequently, TBRs were not in favor for FGS application at the surface of the vulva (Table 2).
- EGFR was moderately expressed in 5/10 dVIN, 11/16 HPV-independent VSCC, 5/15 HSIL and 2/13 HPV-dependent VSCC tissues (Figure 1Q,R), the expression in the remaining samples was low (Figure 1S,T, except 1 HPV-independent VSCC with high expression). HSIL showed a median H-score below 25, the H-scores for other vulvar (pre)malignant tissue types were at least 25 (Table 2).
- EGFR was gradually expressed in healthy vulvar epithelium, being more strongly expressed in the stratum basal compared with the stratum corneum. For (pre)malignant tissues the expression patterns were diverse. Homogenous (Figure 1R), patchy (Figure 1Q,S) and on/off expression patterns (Figure 1T) were observed in these tissues.
- EGFR showed cell membrane staining.

#### 3.2.5. EpCAM—Epithelial Cell Adhesion Molecule

- EpCAM staining was not observed in stromal tissue, except for the endothelial lining of blood vessels. Healthy vulvar epithelium lacked EpCAM expression (Figure 1U). The median H-score of healthy vulvar tissue was not significantly different compared with any vulvar (pre)malignant tissue group (Figure 2), resulting in TBRs < 2, except for dVIN with an TBR of 2.5 (Table 2).
- EpCAM expression was absent or low for all vulvar (pre)malignant tissue types (Figure 1V–Y), with median H-scores below 25 (Table 2).
- No pattern could be recognized due to the low expression of EpCAM in vulvar tissues.
- EpCAM showed cell membrane staining on the endothelial lining of blood vessels.

#### 3.2.6. FRα—Folate Receptor α

- FRα staining was absent in both stromal and healthy vulvar epithelium (Figure 1Z). The median H-score of healthy vulvar tissue was significantly lower compared with the median H-score of dVIN tissue (Figure 2), resulting in a TBR >2. TBRs for other (pre)malignant tissue groups were <2 (Table 2).
- FRα expression was absent or low for all vulvar (pre)malignant tissue types (Figure 1AA–DD), with median H-scores below 25 (Table 2).
- No pattern could be recognized due to the low expression of FRα in all vulvar tissues.
- Cell membrane staining for FRα was observed in lung tumor tissue (control).

#### 3.2.7. MRP1—Multidrug Resistance-Associated Protein

- Low to moderate MRP1 staining was observed in stromal cells and several sebaceous glands of a few healthy and (pre) malignant tissues. No MRP1 expression was observed in healthy vulvar epithelium (Figure 1EE). The median H-score of healthy vulvar tissue was not significantly lower compared with any median H-score of (pre)malignant tissues (Figure 2), resulting in TBRs < 2 (Table 2).
- MPR1 expression was absent or low for all vulvar (pre)malignant tissue types (Figure 1FF–II), with median H-scores below 25 (Table 2).
- No expression pattern could be recognized due to the overall low expression of MRP1.
- In both stromal vulvar tissue as in placental tissue (control), cytoplasmatic and membranous presence of MRP1 was observed on cells.

#### 3.2.8. MUC1—Mucin 1

- Stromal tissue lacked MUC1 staining, except for sebaceous glands positioned in the dermis, which showed moderate or high MUC1 expression (Figure 1JJ). Half of the healthy vulvar epithelial tissues lacked MUC1 expression (Figure 1JJ), others showed low expression restricted to the stratum spinosum. The median H-score of healthy vulvar tissue was significantly lower compared with median H-scores of all vulvar (pre)malignant tissue types (Figure 2), resulting in TBRs > 2 (Table 2).
- Moderate MUC1 expression was observed in 5/10 dVIN (Figure 1 KK), 6/16 HPV-dependent VSCC, 6/14 HSIL and 7/13 HPV-dependent VSCC tissues, the remaining tissues showed low expression (Figure 1LL–NN). Median H-scores for MUC1 expression per vulvar (pre)malignant tissues type were all above 25 (Table 2).
- The expression pattern was heterogenous and patchy throughout all tissue samples.
- MUC1 showed cell membrane staining.

#### 3.2.9. uPAR—Urokinase Plasminogen Activator Receptor

- Low stromal expression of uPAR was observed in healthy and (pre)malignant tissues. Healthy vulvar epithelium lacked uPAR staining(Figure 1OO). The median H-score of healthy vulvar tissues was significantly lower compared with median H-scores of dVIN, HPV-dependent and independent VSCC tissue groups (Figure 2), resulting in TBRs > 2 (Table 2). For the HSIL group, the TBR < 2.
- Moderate uPAR expression was observed in 4/12 HPV-independent VSCC (Figure 1QQ), 1/12 HSIL and 2/12 HPV-dependent VSCC tissues (Figure 1SS), the remaining vulvar (pre)malignant tissues showed low or absent expression (Figure 1PP,RR). Only the median H-score for uPAR expression in the HPV-independent VSCC tissue group was above 25 (Table 2).
- uPAR was heterogeneously expressed throughout (pre)malignant vulvar tissue.
- uPAR showed cell membrane staining and sometimes cytoplasmatic staining in cells.

### 3.3. Evaluation of FGS Criteria

Based on biomarker expression in the vulvar tissue cohort (Section 3.2), only αvβ6 meets all four criteria required to serve as a potential target for tumor-specific imaging in vulvar (pre)malignancies. Therefore, further evaluation of biomarker expression in individual VSCC sections was performed for αvβ6. Representative examples of αvβ6-stained HPV-dependent and HPV-independent VSCC sections, processed by Qupath, are shown in Figures S2 and S3 respectively. The other biomarkers were excluded for further analysis because CAIX, EpCAM, MRP1 showed H-scores for (pre)malignant tissue below 25, TBRs for CD44v6 and EGFR appeared to be inverse and heterogeneous expression was observed for MUC1 and uPAR. 

### 3.4. αvβ6 Expression in Individual VSCC Tissue Sections

**Figure 3:**
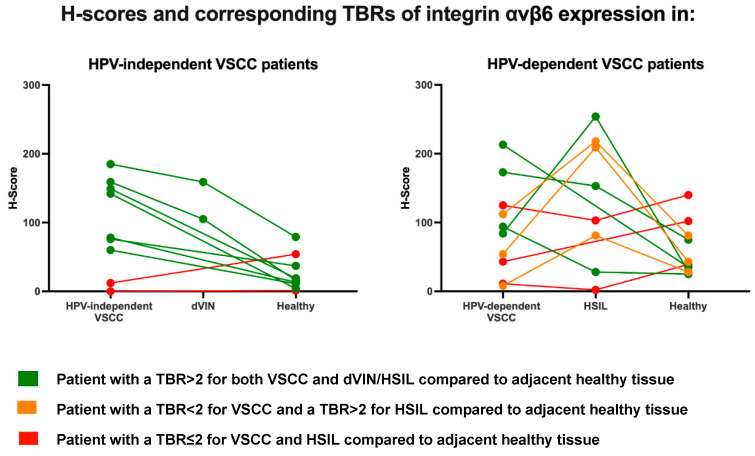
Spaghetti plots of integrin αvβ6 expression within VSCC patients. Lines in this plot represent patients, a dot is an average H-score of a vulvar tissue type within that patient’s tissue section (for instance the average H-score of all adjacent HSIL tissue annotations). Left: HPV-independent VSCC patients, right: HPV-dependent VSCC patients. Based on H-scores, TBRs are calculated (H-score (pre)malignant tissue/H-score of healthy tissue = TBR). A green line indicates higher expression of αvβ6 in (pre)malignant compared to the healthy tissue (TBR > 2); an orange line indicates a TBR < 2 for VSCC and a TBR > 2 for HSIL compared to healthy tissue; a red line indicates higher expression in healthy compared to (pre)malignant tissue (TBR ≤ 1). Not all TBRs of VSCC patients are plotted, as not all tissue samples included normal (and/or premalignant) tissue adjacent to the tumor.

Evaluation of αvβ6 expression on an individual level was not performed for all patients, as 7/16 HPV-independent and 3/13 HPV-dependent VSCC tissue sections did not contain adjacent healthy and/or precursor tissue. In 7/9 HPV-independent and 4/10 HPV-dependent VSCC patients TBRs > 2, based on H-scores of both malignant and premalignant compared with healthy, were observed (represented by a green line, Figure 3). In 3/10 HPV-dependent VSCC sections only favorable TBRs for premalignant compared to healthy were observed (represented by an orange line, Figure 3). In 2/9 HPV-independent and 3/10 HPV-dependent VSCCs TBRs were not in favor of FGS at all (indicated by a red line, Figure 3). Based on this pilot, αvβ6 could serve as a suitable target for tumor-specific imaging in vulvar (pre)malignancies for 78% of HPV-dependent VSCC patients (with adjacent dVIN tissue) and 40% of HPV-dependent VSCC patients. As not all cases showed αvβ6 positivity, CAIX, MUC1 and UPAR TBRS were plotted against αvβ6 TBRs for all 19 above-mentioned VSCC patients, to check case-by-case for an alternative target in case of αvβ6 negativity (Figure S4.) Those alternative targets were chosen as they showed TBRs > 2 for a part of the VSCC patients. In a few cases MUC1 or uPAR might serve as an alternative target (left upper quadrant, Figure S4.)

## 4. Discussion

Demarcation of vulvar (pre)malignancies during diagnosis, staging and surgery is often difficult for clinicians. This phenomenon contributes to significant morbidity and high recurrence rates of up to 40% for VSCC patients after treatment. FGS could improve resection precision of involved tissue and decrease mutilating surgeries. To our knowledge, no data have been published on the use of FGS for VSCC or precursor lesions. Therefore, we used IHC to evaluate target expressions on vulvar tissues to assess their potential for FGS. Our selection of targets was based on (i) enhanced expression in vulvar tumors as described in the available literature and (ii) effectiveness of tracers against these targets obtained from studies with other tumor types [17,18,20,22,23,24].

Out of 9 candidates we assessed, integrin αvβ6 emerged as the most promising target for FGS of VSCC based on immunohistochemistry. This conclusion is based on the upregulated homogeneous expression of αvβ6 in VSCCs compared with surrounding stromal tissue and normal squamous epithelium of the healthy control group. Resulting in TBRs well above the set limit of 2. These suitable TBRs were confirmed within VSCC patient’s using IHC. αvβ6 showed suitable TBRs in 78% of HPV-independent and 40% of HPV-dependent VSCC patients (Figure 3). TBRs for αvβ6 in premalignancies dVIN and HSIL were lower compared with VSCC, but still above the indicated threshold. Higher αvβ6 expression was found in HSIL adjacent to HPV-dependent VSCCs compared with isolated HSIL, encouraging more effective removal of adjacent HSIL during VSCC surgery.

CAIX, EpCAM, MRP1 and FRα showed no or low expression in vulvar malignant tissues and were therefore excluded from further evaluation. CD44v6 and EGFR showed an overall high expression in all tissues, including normal squamous epithelium, resulting in reversed TBRs. If desired, these last two markers could help discriminate infiltrating tumor tissue with FGS, due to the absence of both markers in surrounding stromal tissue. Alternatively, these markers may be used for reversed fluorescence imaging, with distinctive higher expression of the target in healthy compared with malignant tissue [35]. MUC1 and uPAR were excluded from further evaluation due to their heterogenous or patchy expression pattern in vulvar malignant tissue. 

Although several markers (EGFR, CD44v6, MRP1, MUC1 and CAIX) were identified as potential targets for FGS of VSCCs based on our systematic review, their candidacies were not always confirmed in this study [22]. This discordance might be explained by the absence of normal tissue as well as the choice of antibodies and different methods applied in the various IHC studies. Effects of expression patterns per antibody are for example explained in an IHC study on cervical cancer tissues [36]. Finding the antibody with the highest expression pattern in tumor tissue was not the goal of this study. Instead, we chose to apply antibodies most similar to ‘corresponding’ clinically available FGS in order to (i) translate the IHC results in to an imaging setting and (ii) accelerate a future clinical translation if a target/tracer combination showed potential.

Several observations should be considered when proceeding with αvβ6 as a target for FGS in VSCC. First, with the used integrin αvβ6 antibody we observed positively stained sebaceous glands just below the vulvar epithelium in the healthy control tissue. The sac-like alveoli of sebaceous glands are composed of stratified cuboidal or polyhedral epithelial cells. We noticed more sebaceous glands positioned in epithelium of the control vulvar tissues compared with adjacent normal tissue in VSCC samples. The healthy control tissue was obtained from younger women. Sebaceous gland activity is known to decrease in women after menopause, which might be advantageous for TBR-ratios in elderly vulvar cancer patients [37,38]. Whether the remaining positively stained αvβ6 glands lead to difficulties during FGS in younger patients is hard to predict. We assume that the fluorescent signal of these glands will be inferior to the superficial and more enhanced expression in tumors. Another observation that should be considered when proceeding with αvβ6 as target, is the fact that not all VSCC samples showed enhanced αvβ6 expression. Minimal or absent αvβ6 expression was noted in two HPV-independent and two HPV-dependent VSCC samples, resulting in 14% of VSCC cases. In comparison with other squamous tumor, this “on/off” phenomenon was seen in 13% cutaneous squamous cell carcinoma patients [20]. For the αvβ6-negative cases, we assessed if other examined targets could be used instead. Not one of the other examined markers met all four FGS criteria (Section 2.5) and could generally be used for αvβ6-negative cases. However, a case-by-case evaluation should be performed for personalized alternatives in case of αvβ6-negativity. In some cases, MUC1 or uPAR might serve as an alternative VSCC-target for FGS in case of αvβ6 negativity (Figure S4, left upper quadrant).

An explanation for the different expression patterns of integrin αvβ6 in VSCC remains elusive. A hallmark function of αvβ6 is the activation of transforming growth factor-β1 (TGF-1) to modulate innate immune surveillance in e.g., skin. Therefore, it is possible that different expression patterns of αvβ6 are explained by the difference in tumor-immune infiltration [38]. In addition, different FIGO stages of included tumors may explain variation in expression patterns. High-grade progressive tumors show different levels of cell-adhesion compared with low-grade tumors and integrins are cell surface receptors responsible for cell-to-matrix and cell-to-cell adhesion [38,39,40]. Structural differences in expression patterns, especially those observed between the virally and non-virally induced tumors, should be confirmed in larger cohort studies. The availability of patients’ medical history including FIGO stage, detailed demographics, surgical margins, and other characteristics could improve the value of the data set substantially. Especially the assessment of whether αvβ6 is overexpressed in associated locoregional lymph node metastases compared with background tissue and negative lymph nodes. If TBRs for involved lymph nodes are applicable, FGS positive nodes could improve overall survival [41]. In addition, future research should investigate whether adjuvant radiotherapy, as part of treatment for locally advanced and metastatic disease, is of influence on αvβ6 expression patterns.

As indicated above, evaluation of αvβ6 as a target for FGS of vulvar (pre)malignancies was chosen based on promising (pre)clinical results in other cancer types [20]. The benefit of this is the availability of imaging agents targeting αvβ6, like the recently developed linear peptide A20FMDV2 or knottin-peptide R01-MG [42,43]. For FGS application the latter peptide was conjugated to fluorescent tracer IRDye800CW to improve complete resection of patients with pancreatic ductal adenocarcinoma. In subcutaneous and orthotopic mouse pancreatic tumor models R01-MG-IRDye800 showed specific targeting to αvβ6 and holds promise as a tool to recognize pancreatic cancer with FGS [42]. Another example of a fluorescent imaging agent is cRGD-ZW800-1, that binds primarily to αvβ3 but has also affinity for αvβ6. This agent is already assessed for clinical use in colorectal cancer imaging [44]. No data can be found on αvβ3 expression in vulvar tissue. Therefore, the potential of this imaging agent for FGS in VSCC should be further investigated. 

Although this paper focused on immunohistochemical evaluation of molecular imaging targets meant for FGS, αvβ6 could also be used as target for imaging with PET or SPECT/CT [45,46]. In addition, αvβ6 could be used as target for different treatment modalities. It’s suitability for this purposes indicated by several anticancer strategies based on αvβ6 targeting, including immunoliposomes used as vectors in tumor targeted therapy [47]. Future research should focus on evaluation of αvβ6-targeting probes in ex-vivo models, for instance in 3D vulvar skin-tumor models, as a step towards clinical translatability [48,49]. Presuming that targets expressed on VSCC-cells are mostly unoccupied in-situ, so that an αvβ6-targeted FGS probe will be able to access the ligand binding site. Besides multiple deployment of this αvβ6 target, it would also be nice to verify in future studies if the modality FGS on itself might be useful for other vulvar diseases, as e.g., Paget disease of the vulva, which often extends beyond the visible lesion.

Next to the limited size of the cohort a limitation of this study is the pre-selection of biomarkers. The selection was based on enhanced expression of targets as described in the available literature and effectiveness of tracers against these targets obtained from studies with other tumor types [17,18,20,22,23,24]. Alternatively, an ‘omics’ search could be performed in combination with artificial intelligence to detect all currently (un)known targets and check their expression on vulvar healthy compared to (pre)malignant tissues. Nevertheless, promising target/tracer combinations based on ‘omics’ findings should still be evaluated in the clinic. Another limitation is the slight overestimation of Qupath for epithelial cells in stromal areas, which was observed in most tissue sections. As this phenomenon was equally observed in all differently selected tissue types, we assessed that it does not affect the tumor-to-background ratio substantially. It might even underestimate this ratio as almost all stromal cells stained negative. In addition, the semi-automated analysis using Qupath on small cohorts is labor intensive, even compared to visual scoring of IHC-stained sections. It is therefore desirable to further optimize this method, before testing the potential of targets for IGS application on a larger cohort of tissues. A self-learning algorithm can be drawn based on this training set for future reference. Furthermore, since this study was limited by scarcity of vulvar (pre)malignant tissues, it was not possible to define the sensitivity and specificity for IGS suitability per marker. However, this set could be used as a ‘training set’ for a future multicenter study wherein sufficient vulvar tissue samples can be included. 

## 5. Conclusions

αvβ6 is a promising target for tumor-specific (pre- and intra-operative) molecular imaging of VSCC lesions, which can be hard to distinguish from healthy tissue. For HPV-unrelated VSCCs with adjacent dVIN, that comprise the vast majority of all VSCCs, αvβ6 has shown great potential for precise discrimination at the superficial tissue margins. Further research is needed to validate the use of an αvβ6-targeted probe for FGS of vulvar (pre)malignancies. Finally, it should be verified whether addition of this technique leads to fewer recurrences and surgery-related morbidities for VSCC patients.

## Figures and Tables

**Table 1:** Antibodies used for immunohistochemical analysis, including source, clone, stock, dilutions, antigen retrieval applied and source of control tissue per biomarker tested.

Biomarker	Source	Clone Number	Catalogue Number	Stock	Dilution	Antigen Retrieval	Positive Control
αvβ6	Biogen, Inc., Cambridge, MA, USA	6.2A1	62A1CEO2	50 µg/mL	1/100	0.4% pepsin (S3002 Agilent) 37 °C for 15 min.	Normal colon
CA IX	Santa Cruz Biotechnology, Inc., Danvers, MA, USA	H-11	Sc-365900	200 µg/mL	1/2500	Target retrieval solution, pH 6.1 (K8005 Agilent)	Normal stomach
CD44v6	Abcam, Cambridge, UK	VFF7	ab30436	1 mg/mL	1/3200	Target retrieval solution, pH 6.1 (K8005 Agilent)	Normal skin
EGFR	Dako, Glostrup, Denmark	E30	M7239	286 µg/mL	1/600	0.4% pepsin (S3002 Agilent) 37 °C for 10 min.	Normal placenta
EpCAM	LUMC, department of pathology ^1^	323/A3	-	0.4 mg/mL	1/1600	0.1% trypsin (T7409 Sigma Aldrich) 37 °C for 30 min.	Colon tumor
FRα	BioCare Medical, Pacheco, CA, USA	26B3.F2	BRI 4006K AA (kit)	Assay kit	N.A.	Ready-to-use	Lung tumor
MRP1	Santa Cruz Biotechnology, Inc., Danvers, MA, USA	QCRL-1	Sc-18835	200 µg /mL	1/400	Target retrieval solution, pH 6.1 (K8005 Agilent)	Normal placenta
MUC1	Invitrogen, Waltham, MA, USA	E29	MA5-14077	0.2 mg/mL	1/4800	Target retrieval solution, pH 9.0 (K8004 Agilent)	Normal colon
uPAR	Monopar ^2^	ATN617	-	0.48 mg/mL	1/200	Target retrieval solution, pH 6.1 (K8005 Agilent)	Colon tumor
p16	Roche, Almere, The Netherlands	E6H4	06695248001	Ready-to-use	1/25	TRIS/EDTA	Normal cervix
p53	DAKO, Santa Clara, CA, USA	DO-7	GA61661-2	Ready to use	1/2000	TRIS/EDTA	Normal cervix

^1^. Kindly provided by Jaap van Eendenburg, department of pathology LUMC, The Netherlands. ^2^. Kindly provided by Andrew Mazar, Monopar Therapeutics Inc., Wilmette, IL, USA.

**Table 2:** Median, minimum (min), maximum (max) H-scores and tumor-to-background ratios (TBRs) for each marker per vulvar tissue group are presented. TBRs > 2 are displayed in green (IGS criterion 1, Section 2.5) and a minimum median H-scores of at least 25 in blue (IGS criterion 2, Section 2.5). HPV− VSCC = HPV-independent VSCC and HPV+ VSCC = HPV-dependent VSCC.

	αvβ6				CAIX				CD44v6			
	Median	Min	Max	TBR	Median	Min	Max	TBR	Median	Min	Max	TBR
**Healthy (*n* = 15)**	9	2	31	-	2	0	19	-	240	173	283	-
**dVIN (*n* = 10)**	**59 ^b^**	20	216	**6.6**	7 ^b^	1	65	**3.5**	**248**	123	275	1.0
**HPV− VSCC (*n* = 16)**	**118**	0	231	**13.1**	9 ^a^	1	77	**4.5**	**196**	96	271	0.8
**HSIL (*n* = 15)**	**42 ^b^**	3	145	**4.7**	5	1	92	**2.5**	**223**	119	279	0.9
**HPV + VSCC (*n* = 13)**	**93**	7	213	**10.3**	6	1	102	**3.0**	**118 ^a^**	51	267	0.5
	**EGFR**				**EpCAM**				**FRα**			
	Median	Min	Max	TBR	Median	Min	Max	TBR	Median	Min	Max	TBR
**Healthy (*n* = 15)**	61	2	200	-	2	0	12	-	3	1	43	-
**dVIN (*n* = 10)**	**40**	2	138	0.7	5	0	11	**2.5**	1	1	3	0.3
**HPV− VSCC (*n* = 16)**	**129**	4	253	**2.2**	3	0	17	1.5	4 ^a^	2	7	1.3
**HSIL (*n* = 15)**	10	0	213	0.2	2	0	17	1.0	2	0	28	0.7
**HPV + VSCC (*n* = 13)**	**25**	5	106	0.4	7 ^a^	0	91	3.5	4	2	60	0.3
	**MRP1**				**MUC1**				**uPAR**			
	Median	Min	Max	TBR	Median	Min	Max	TBR	Median	Min	Max	TBR
**Healthy (*n* = 15)**	4	0	29	-	11	1	81	-	6	1	22	-
**dVIN (*n* = 10)**	8	0	24	2.0	**63**	20	206	**5.7**	12 ^b^	2	35	2.0
**HPV− VSCC (*n* = 16)**	13 ^c^	0	42	0.3	**34 ^a^**	3	184	**3.1**	**37 ^c^**	5	91	**6.2**
**HSIL (*n* = 15)**	2	0	22	0.5	**40**	3	201	**3.6**	6 ^c^	0	73	1.0
**HPV + VSCC (*n* = 13)**	2	0	17	0.5	**62**	14	176	**5.6**	19 ^a^	1	125	**3.1**

^a^ = 1 tissue section missing, ^b^ = 2 tissue sections missing, ^c^ = 3 tissue sections missing.

## Data Availability

The study did not report this type of data.

## Supplementary Materials

The following are available online at https://www.mdpi.com/article/10.3390/cancers13236006/s1, Table S1: Antigen retrieval methods applied: pepsin, trypsin and DAKO citrate buffers; Script S1: Script used for digital pathology image analysis using Qupath; Figure S1: Zoomed images of immunohistochemical control staining’s of nine examined markers, Figure S2: By Qupath processed tissue sections of a patient with HPV-dependent VSCC, Figure S3: By Qupath processed tissue sections of a patient with HPV-independent VSCC with tissue transition zones, Figure S4: Comparison of MUC1, CAIX and uPAR TBRs against αvβ6 TBRs for VSCC patients.

## Abbreviations

αvβ6—integrin alphavbeta6, CAIX—carbonic anhydrase IX, CD44v6—CD44 variant 6, EGFR—epidermal growth factor receptor, EpCAM—epithelial cell adhesion molecule, FRα—folate receptor α, MRP1—multidrug resistance-associated protein, MUC1—mucin 1, uPAR—urokinase plasminogen activator receptor.
