# Supplementary material for: Integrin αvβ6 as a Target for Tumor-Specific Imaging of Vulvar Squamous Cell Carcinoma and Adjacent Premalignant Lesions
Source: Cancers (Basel). 2021 Nov 29;13(23):6006. doi: 10.3390/cancers13236006 (PMC8656970; doi:10.3390/cancers13236006)
Supplement: Supplementary file 1 [file cancers-13-06006-s001.zip › cancers-1459464-supplementary.pdf]

cancers

MDPI

Article

Integrin αvβ6 as a Target for Tumor-Specific Imaging of Vulvar Squamous Cell Carcinoma and Adjacent Premalignant Lesions

Bertine W. Huisman, Merve Cankat, Tjalling Bosse, Alexander L. Vahrmeijer, Robert Rissmann, Jacobus Burggraaf, Cornelis F. M. Sier and Mariette I. E. van Poelgeest

Table S1. Antigen retrieval methods applied: pepsin, trypsin and DAKO citrate buffers.

Antigen retrieval	Method
**Pepsin**	2.5 ml 1M HCL was added to 1 gram pepsin (DAKO LOT#10145658) and supplemented with 250 mL demineralized water. After a washing step the slides were placed in this solution and incubated in a water bath at 37°C for 20 minutes. Thereafter, slides were washed in demineralized water and PBS.
**Trypsin**	0.5gram Trypsin (Sigma T-7409 LOT#SLCD1173) was added to 0.5 gram Calcium chloride and supplemented with 250mL demineralized water. The pH of this solution was adjusted to 7.4. After a washing step the slides were placed in the pre-heated solution and incubated in a water bath at 37°C for 30 minutes. Thereafter, slides were washed in demineralized water and PBS.
**Citrate buffer pH 6.0**	The slides were in a PT-link (DAKO) with citrate buffer (pH 6.0) heated to 65°C and 95°C for a short period. Thereafter, slides were cooled to 65°C and washed in demineralized water and PBS.
**Citrate buffer pH 9.0**	Slides were in a PT-link (DAKO) with citrate buffer (pH 9.0) heated to 65°C and 95°C for a short period. Thereafter, slides were cooled to 65°C and washed in demineralized water and PBS.

```
setColorDeconvolutionStains('{"Name" : "H-DAB modified", "Stain 1" : "Hematoxylin", "Values 1" : "0.402 0.593 0.698", "Stain 2" : "DAB", "Values 2" : "0.26 0.491 0.831", "Background" : " 252 253 249 "}'); selectAnnotations() runPlugin('qupath.imagej.detect.cells.WatershedCellDetection', '{"detectionImageBrightfield": "Hematoxylin OD", "requestedPixelSizeMicrons": 0.38895, "backgroundRadiusMicrons": 8.0, "medianRadiusMicrons": 0.0, "sigmaMicrons": 1.5, "minAreaMicrons": 10.0, "maxAreaMicrons": 400.0, "threshold": 0.1, "maxBackground": 2.0, "watershedPostProcess": true, "excludeDAB": true, "cellExpansionMicrons": 5.0, "includeNuclei": false, "smoothBoundaries": true, "makeMeasurements": true}'); runPlugin('qupath.imagej.detect.cells.PositiveCellDetection', '{"detectionImageBrightfield": "Hematoxylin OD", "requestedPixelSizeMicrons": 0.38895, "backgroundRadiusMicrons": 8.0, "medianRadiusMicrons": 0.0, "sigmaMicrons": 1.5, "minAreaMicrons": 10.0, "maxAreaMicrons": 400.0, "threshold": 0.1, "maxBackground": 2.0, "watershedPostProcess": true, "excludeDAB": true, "cellExpansionMicrons": 5.0, "includeNuclei": false, "smoothBoundaries": true, "makeMeasurements": true, "thresholdCompartment": "Cell: DAB OD mean", "thresholdPositive1": 0.2, "thresholdPositive2": 0.4, "thresholdPositive3": 0.6000000000000001, "singleThreshold": true}');
```

Scheme S1. Script used for digital pathology image analysis using Qupath. The script includes cell detection, positive cell decection and cell classification.

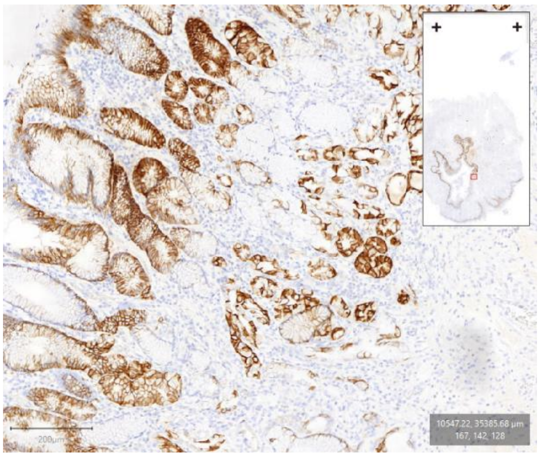

αvβ6 – normal colon

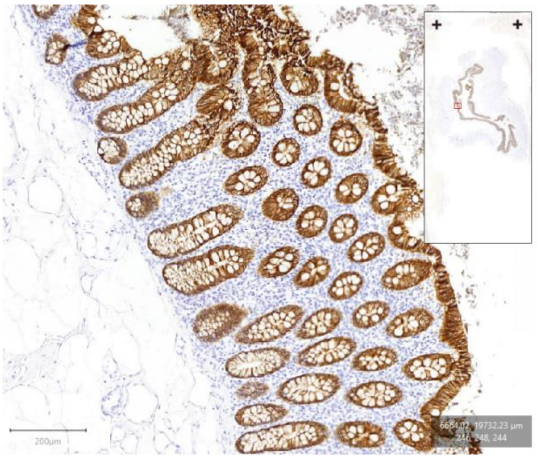

CAIX – normal stomach

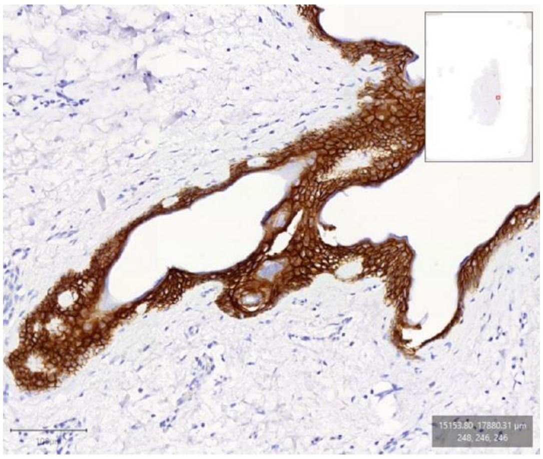

CD44v6 – normal skin

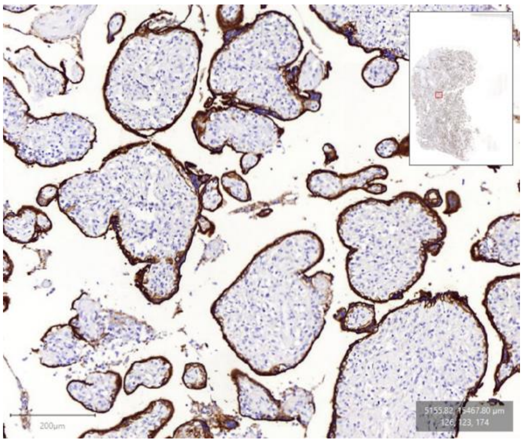

EGFR – normal placenta

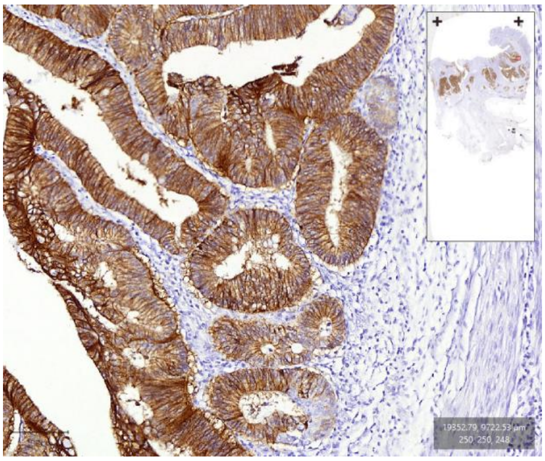

EpCAM – colon tumor

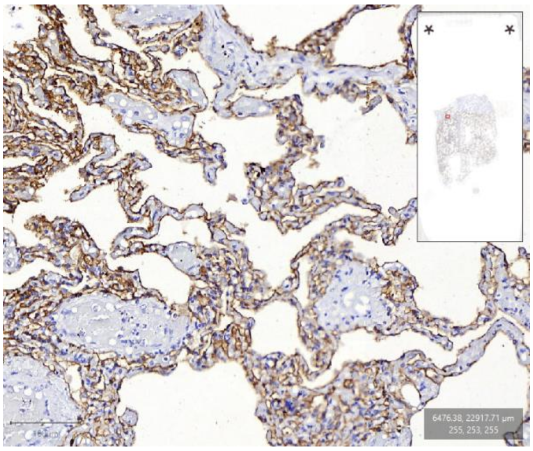

Folate receptor alpha – lung tumor

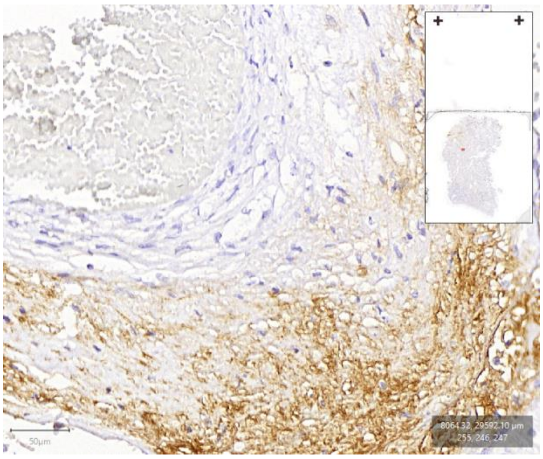

MRP1 – normal placenta

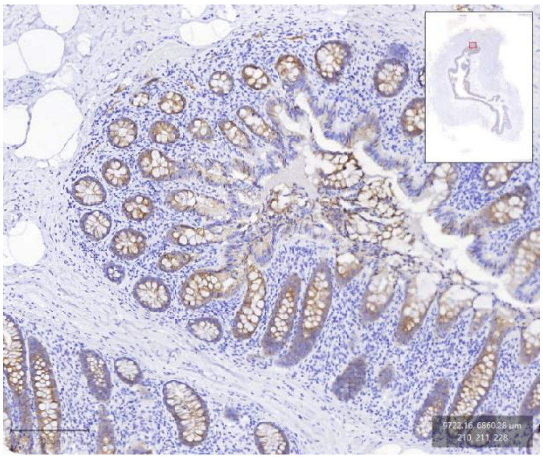

MUC1 – normal colon

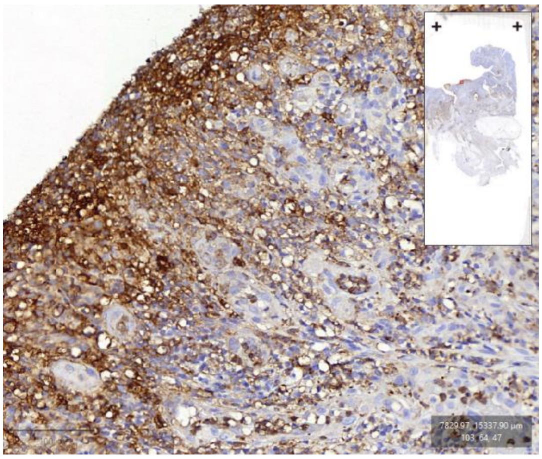

uPAR – colon tumor

Figure S1. Zoomed images of immunohistochemical control staining's of nine examined markers. The titles of the images indicate the marker/target followed by the examined control tissue. In the right upper corner of the zoomed image, an overview of the whole tissue section is shown. Scale bars indicate 50, 110 or 200 µm.

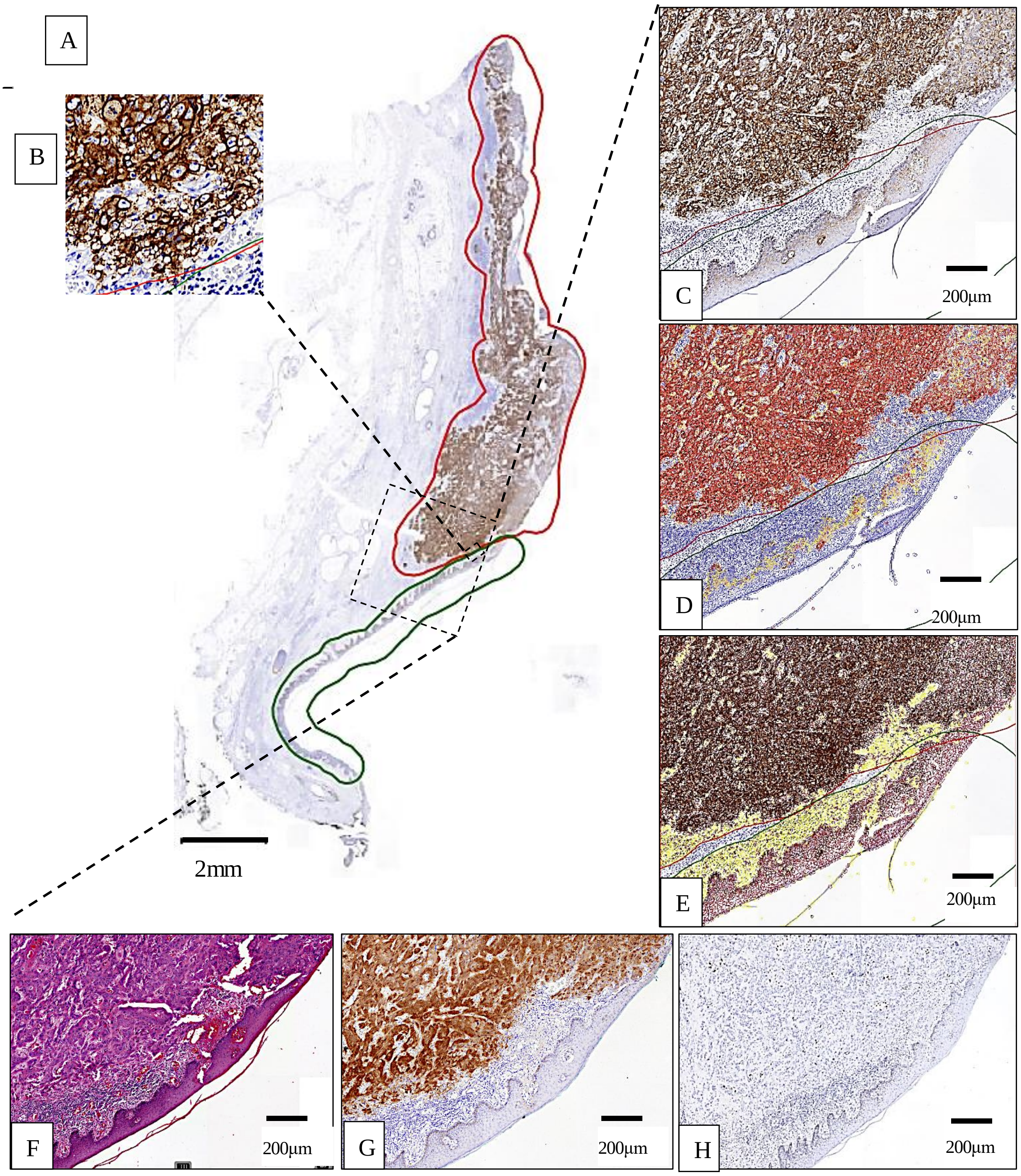

Figure S2. By Qupath processed tissue sections of a patient with HPV-dependent VSCC: A. Whole tissue section with integrin αvβ6 DAB staining, an insert (B) shows tumor cells at a higher magnification, C. Magnification of the tissue section as shown in A, D. Positive cell detection markup image of Qupath on the same tissue section as shown in A, with epithelial cells colored according to integrin αvβ6 expression intensity: blue=absent staining; yellow=low intensity (1+); orange=moderate intensity (2+); red=high intensity (3+), E. Classification markup image of Qupath on the same tissue section as shown in A, with cells colored according to their tissue class: (dark)red=cells defined as 'epithelial'; yellow=non-epithelial cells defined as 'rest', e.g. stromal or immune cells. In image A, B, C, and D tissue fragments are annotated: green annotation=normal/healthy; red annotation=HPV-related VSCC. F. Sequential tissue section with HE staining, G. sequential tissue section with P16 staining, H. sequential tissue section with P53 staining. Scale bars represent 2mm (A) and 200µm (B-H).

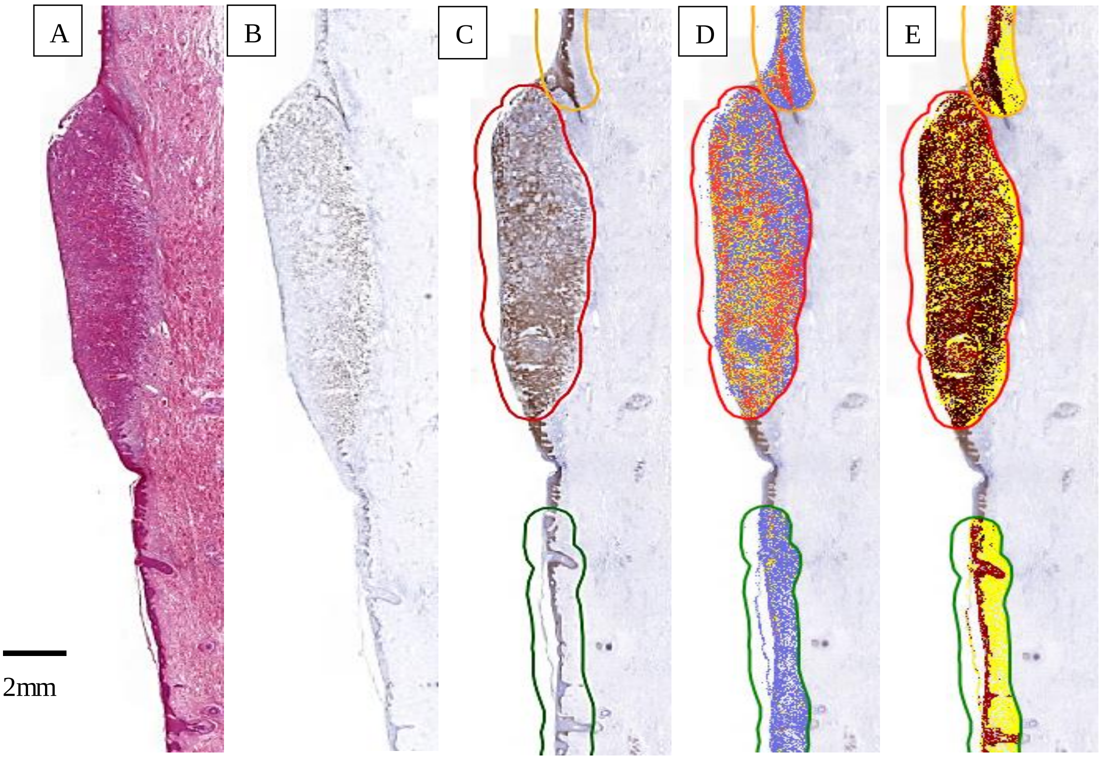

Figure S3. By Qupath processed tissue sections of a patient with HPV-independent VSCC with tissue transition zones: A. HE staining, B. P53 staining, C. integrin αvβ6 DAB staining, D. Positive cell detection markup image of Qupath on the same tissue section as shown in C, with epithelial cells colored according to integrin αvβ6 expression intensity: blue = absent staining; yellow = low intensity (1+); orange = moderate intensity (2+); red = high intensity (3+), E. Classification markup image of Qupath on the same section as shown in C, with cells colored according to their tissue class: (dark)red = cells defined as 'epithelial'; yellow = non-epithelial cells defined as 'rest', e.g. stromal or immune cells. In image C, D and E tissue fragments are annotated: green annotation = normal/healthy; orange annotation = premalignant dVIN; red annotation = HPV-related VSCC.). In the transition zone between normal vulvar epithelium and HPV-dependent VSCC tissue an annotation is lacking, as the pathologist found it difficult to classify this tissue. Scale bar represents 2mm.

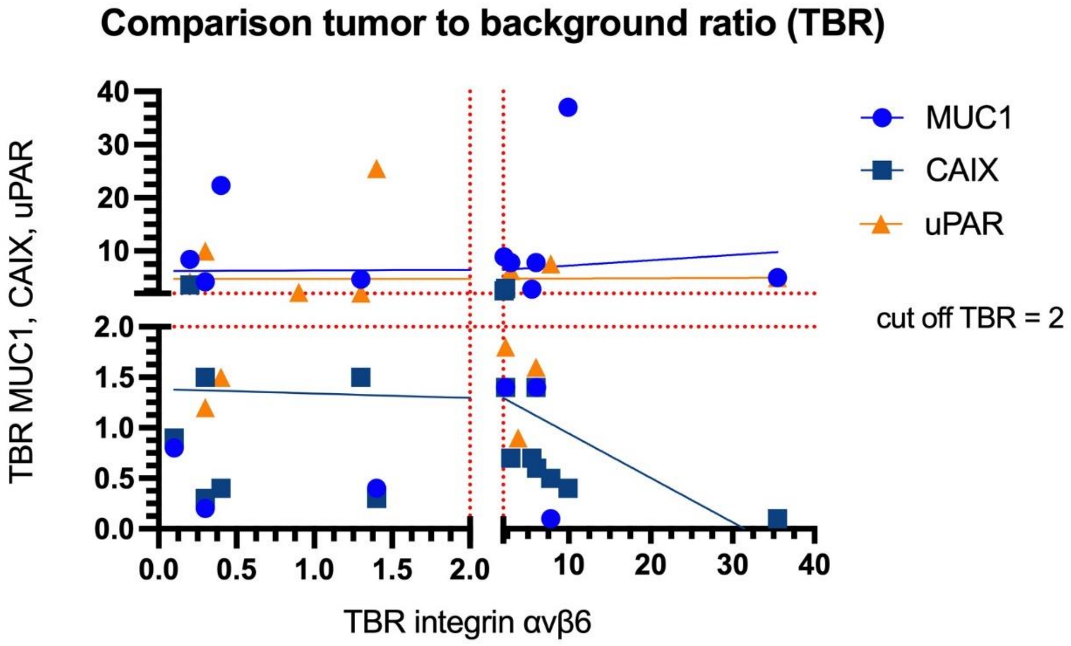

Figure S4. Tumor to background ratios (TBRs) of MUC1, CAIX and uPAR compared to TBRs of integrin αvβ6 for 19 VSCC patients. TBRs were calculated: 'H-score VSCC/H-score healthy', within a VSCC tissue section. Occasionally a staining of a tissue slide was missing and could not be scored. The left upper quadrant shows the cases in which integrin αvβ6 is negative, and one of the other markers might serve as an alternative target for FGS in VSCC.
